# An assessment of the effect of user fee policy reform on facility-based deliveries in rural Zambia

**DOI:** 10.1186/s13104-016-2316-8

**Published:** 2016-12-07

**Authors:** Chitalu Miriam Chama-Chiliba, Steven Fredric Koch

**Affiliations:** 1 Department of Economics, University of Zambia, Lusaka, Zambia; 2 Department of Economics, University of Pretoria, Pretoria, South Africa

**Keywords:** Facility-based deliveries, Rural, User fees, Difference-in-differences, Maternal health, Zambia

## Abstract

**Background:**

Improving maternal health outcomes by reducing barriers to accessing maternal health services is a key goal for most developing countries. This paper analyses the effect of user fee removal, which was announced for rural areas of Zambia in April 2006, on the use of public health facilities for childbirth.

**Methods:**

Data from the 2007 Zambia Demographic and Health Survey, including birth histories for the five years preceding the survey, is linked to administrative data and geo-referenced health facility census data. We exploit a difference-in-differences design, due to a differential change in user fees at the district level; fees were removed in 54 rural districts, but not in the 18 remaining urban districts. We use multilevel modelling to estimate the effect of this policy change, based on 4018 births from May 2002 to September 2007, covering a period before and after the policy announcement in April 2006.

**Results:**

The difference-in-difference estimates point to statistically insignificant changes in the proportion of women giving birth at home and in public facilities, but significant changes are found for deliveries in private (faith-based) facilities. Thus, the abolition of delivery fees is found to have some effect on where Zambian mothers choose to have their children born.

**Conclusion:**

The removal of user fees has not overcome barriers to the utilisation of delivery services at public facilities. User fee removal may also yield unintended consequences deterring the utilisation of delivery services. Therefore, abolishing user fees, alone, may not be sufficient to affect changes in utilisation; instead, other efforts, such as improving service quality, may have a greater impact.

## Background

Zambia’s maternal mortality rate (MMR) of 591 per 100,000 live births in 2007 [1]—the MMR estimates for 1996 and 2001 were 649 and 729, respectively [2]—suggests stagnation, at best, implying that the risk of death during childbirth remains high. Despite this risk, more than 67% of Zambian women in rural areas deliver at home [1]. Home delivery is assumed to be a maternal mortality risk, because of its positive association with maternal mortality [3] (although more recent research is needed [4]) and the negative association between skilled assistance at birth and maternal mortality [5]. Although safe motherhood strategies [5] do not preclude home delivery, fewer than 50% of births in Zambia are attended to by a skilled assistant [6], implying that home delivery remains riskier. To reduce MMR through reductions in home deliveries, many sub-Saharan African countries abolished or reduced health service user fees [7–9], or exempted groups from payment requirements [10, 11]. The expected effect, an inverse relationship between delivery service user fees and delivery service utilisation as implied by the economic law of demand [10–13], has been uncovered, although not uniformly [7]. Two systematic reviews of user fee abolition argue that there is a dearth of robust evidence supporting the hypothesis that user fee reductions raise utilisation [14, 15].

The existing literature, however, has generally ignored other characteristics, when considering user fee removal effects [16]. Quality of care, one characteristic expected to influence maternal health service utilisation is rarely captured in household surveys. The same is true of cost-shifting; in the absence of user fees, women have been required to purchase supplies normally provided by a health facility (e.g., bleach, gloves and sanitary pads), when admitted for delivery services [17, 18], or arrange for their own food [18]. Omitting this information may give an incomplete picture of user fee effects, and lead to erroneous conclusions. Although it is not possible to address each of these concerns, we are able to capture some aspects of care quality, and include those in the analysis.

We use data from Zambia to analyse the effect of user fee abolition, focussing our attention on a woman’s decision to give birth at home, at a public health facility or at a private facility. The analysis is built on a difference-in-difference design estimated via multilevel logistic regression. There are two levels accounting for both individual and household measures, as well as health district characteristics. The results suggest that user fee abolition has not encouraged women to make increased use of public health facilities for childbirth, and, therefore, it is concluded that other barriers remain that deter women.

### User fee policy context

User fees in Zambia were abolished in public health facilities providing primary health care services. The policy change took effect in April 2006 in 54 rural districts, but not in the remaining 18 urban health districts. In terms of organisation, the public health care system in Zambia is a three level structure consisting of primary, secondary and tertiary levels. The primary health care level facilities, in order of increasing level of service delivery, are health posts, health centres and districts hospitals. The secondary level hospital, also known as provincial referral hospitals, are the highest referral hospital in a province while the tertiary hospitals are the highest level of care, offering the both specialised and teaching services. In addition, health services are provided by four main players namely the government, faith-based not-for-profit providers, private-for-profit providers and traditional practitioners. The public sector is the biggest health provider with about 90% of patients seeking health care services from facilities that are government owned.

Free health services, as announced at that time, included all aspects of preventative and curative services at Health Posts and Health Centres, including Hospital Affiliated Health Centres. Patients referred to first level (district) hospitals were to be treated free of charge for all services at such facilities, and upward referrals were also user fee free. Prior to the policy change, preventative services, such as antenatal care, family planning and counselling, were already exempt from payment; delivery services were not. Instead, health providers in various regions set fees, approved by the Ministry of Health [8]. Delivery fees at public health facilities varied from K10, 000 ($3) to K30, 000 ($9) [28, 29], despite the fact that the $1.25 per day headcount poverty ratio in Zambia was 68.5% in 2006 [30].

### Data

The data comes from the Zambia Demographic and Health Survey (ZDHS), the Zambia Health Facility Census (ZHFC) and the Ministry of Health’s (MoH) Health Management and Information Health System (HMIS). The ZDHS is a nationally representative survey that interviewed 7146 women aged 15–49; the data was collected between April–October 2007 [1]. Given the ZDHS geo-reference information, it is possible to place each respondent within a cluster, which is within an administrative district. The distance to the nearest health facility was obtained by overlaying the ZDHS geo-reference information with geo-reference data from the 2005 ZHFC covering public, faith-based (also known as mission or non-governmental organisation) and larger private-for-profit health facilities in the country. From the overlay, straight-line distances—the centre of ZDHS cluster to nearest health facility—were calculated with ArcGIS.

The ZDHS contains birth histories on 5410 children, born between May 2002 and September 2007, (only the most recent birth was considered). Considering respondents with complete maternal covariate information; including the mother’s partner’s characteristics and other community level variables reduces the sample to 4018, with 2368 births occurring before the policy change and 1650, while 2373 mothers from fee abolished (rural) districts form the treatment group and 1646 mothers from fee paying (urban) districts form the control group. We expect that a reduction in user fees, ceteris paribus, reduces the cost of delivery and may also lead to more births at the health facility. Fertility rates in Zambia are among the highest in the sub-Saharan Africa region, at 6.2 in 2007, and even higher in rural areas. More so, between 2001 and 2002 and 2007, fertility rates in rural areas increased from 6.9 to 7.5, while the rate in urban areas remained constant at 4.3.

#### Household variables

Explanatory variables, described in Table 2, are informed by previous research. Higher birth order women may not seek maternal health care, due to knowledge and experience gained from past births, limited care available for younger children and negative comments received at the health facility [16]. The woman’s age was omitted due to high correlation (0.8) with parity [19]. However, additional analysis that included age rather than parity was also done and the results obtained were similar to those obtained using parity. Education, the woman’s and her partner’s, is expected to increase health facility utilisation [19, 20], as is socio-economic status—captured by a wealth index categorized as poor, middle and rich. Even though user fees were removed in Zambia, wealthier households remain better equipped to cope with other direct and indirect costs of care [7]. Also, religious attitudes may affect choices [7, 21]. Finally, we address residential stability by including duration at current residence [19].

In addition to the preceding household variables, experiences matter. Receiving ante natal care (ANC) during pregnancy engenders familiarity with the health system and facility, providing opportunities for health workers to promote delivery services [16]. The quality of ANC received signals the likely quality of delivery services. Thus, we construct a composite quality index based on the essential ANC components of focussed ANC [22]. Our index, modelled via multiple correspondence analysis (MCA), captures ANC at a facility; therefore, it serves as a proxy for the expected quality of delivery services. The MCA includes: attendance by skilled health worker, weight and height measured, blood pressure checked, urine and blood sample taken, told about pregnancy complications, given or bought iron tablets, and received fansidar as prophylaxis for malaria prevention. Although principal component analysis (PCA) is widely used [19, 23], MCA is designed for categorical variables [24]. The composite index score for each woman was calculated from the generated weights in Table 1; higher and lower quality of care are reflected in positive and negative values, respectively.

**Table 1:** MCA variables and weights associated with quality of ANC

Variable	Categories	Weights
Skilled assistance	Attended by skilled worker during visit	0.171
	Not attended by skilled worker during visit	−2.897
Weight	Weighed during pregnancy	0.346
	Not Weighed during pregnancy	−3.238
Height	Height measured during pregnancy	1.555
	Height not measured during pregnancy	−0.535
Blood pressure	Blood pressure checked during pregnancy	0.634
	Blood pressure not checked during pregnancy	−2.864
Urine sample	Urine sample taken during pregnancy	2.363
	Urine sample not taken during pregnancy	−0.601
Blood sample	Blood sample taken during pregnancy	1.218
	Blood sample not taken during pregnancy	−1.792
Complications	Told about complications during pregnancy	0.516
	Not told about complications during pregnancy	−1.514
Iron tablets	Given or bought iron tablets during pregnancy	0.079
	Not given or bought iron tablets during pregnancy	−1.105
Prophylaxis	Took fansidar as prophylaxis for malaria prevention in pregnancy	0.209
	Did not take fansidar as prophylaxis for malaria prevention in pregnancy	−1.829

Composite index variables, categories and weights from the first MCA dimension with iterative adjustment based on the ‘mca’ command in STATA 12. These are adjusted for the principal inertias, and the first dimension explained 64% of total inertia

#### Community variables

For community-level data, ZDHS clusters are linked to district-level HMIS data. A *ZDHS* cluster is representative of an enumeration area with an average size of 130 households, or 600 people [25]. The ZDHS geo-referenced data is linked to each cluster and was used to identify the specific district covered during the survey. The district-level HMIS data include monthly information on supply and use of a wide range of services by all public health facilities nationwide, aggregated to the district level. Therefore, all the community level variables were captured at district level. Living closer to a facility is expected to increase maternal health service utilisation, due to lower direct and indirect transport costs [16, 19]. Objective measures of facility quality, like drug availability, are essential determinants of delivery services [16]. Poverty status in the community, also captured, follows the material deprivation index (MDI). The MDI ranges from −4.65, least deprived, to 1.66, most deprived [26]. Community ANC uptake is also included, as it is expected to relate to delivery service utilisation [19, 20, 27]. Finally, traditional birth attendants (TBA) in developing countries cannot be ignored [1].

## Methods

One year after the user fee abolition announcement, the 2007 Zambia Demographic and Health Survey (ZDHS) was instituted. Given the birth histories contained in the ZDHS, we generate a dataset of births that occurred before fee abolition in April 2006 and after. Geocoded ZDHS household responses are linked to health facility census data, as well. Thus, the captured data underpins a quasi-experimental difference-in-difference (DID) design.

To assess the effect of the abolition of user fees on the place of delivery, we apply DID, exploiting the staggered nature of the policy implementation. The DID regression is estimated by a (two-level) multilevel logistic regression, which accounts for not only the hierarchical structure of the data, but also enables the estimation of community level effects on the outcome variables [19]. Ignoring observation clustering yields underestimated standard errors, and may result in spurious significant results [19, 31].

The two-level multilevel logistic regression models used in this study consist of two sub-models at Level 1 and 2, were used. Level 1 represents the relationships among the individual-level variables, while level 2 examines the influence of community-level factors. Both individual and household characteristics are individual level variables, because the average number of women in a household is too small for the identification of a further level of analysis. To assess the influence of unobserved community level characteristics on the overall variation in the use of delivery services, we specify a null model (without covariates). Two extended model specifications are also fitted; they examine potential determinants of the probability of delivery at a specific type of facility. Model 1 includes individual characteristics, only, while Model 2 includes both individual-level and community-level variables. Statistical analyses are carried out using xtlogit in Stata 12 [32].

The model is specified as follows:

$$\eqalign{ & Logit\left( {{\pi _{ij}}} \right) = {\beta _0} + {\beta _1}{D_{2006}} + {\beta _2}{D_{region}} \cr & \qquad \qquad \quad + \,{\beta _3}{D_{2006}} \times {D_{region}} + {\beta _4}{x_{ij}} + {\beta _5}{z_j} + {\upsilon _j}, \cr}$$

where $\pi_{ij}$ is the probability that the delivery occurs in that specific location for woman *i* in community *j*. There are three separate binary dependent variables used in the analysis: delivery at home, delivery at a public health facility and delivery in a private health facility; including them together as one categorical variable in a multinomial model does not change the conclusions reported here, and results are available upon request. Due to the small proportions, we combined two categories: delivery in faith-based organisations and private health facilities. In Zambia, the faith-based hospitals are supported by both government and other agencies, and are mostly run as non-profit organisations. Thus, we specify a logit model to estimate the probability that a woman will choose to deliver at home, at a private health facility or public health facility. The reference categories for home and private deliveries are restricted to include only deliveries in public health facilities, given that a priori, the policy may not only lead to a shift from home to public births, but also from private to public births and vice versa.

We include a dummy for region, *D*_*region*_, (rural/urban) and time, *D*_2006_, (before/after policy change) and their interaction, *D*_2006_ × *D*_*region*_. The vectors *x* and *z* represent individual and community level variables, respectively. We include, in *x*, variables that the duration of residence, to address selective migration and residential stability, while *ν*_*j*_ ∼ *N*(0, *σ* ^2^) represents the random effects for the *jth* community. The time dummy captures constant-across-region aggregate factors influencing a women’s delivery choice. The region dummy captures constant-across-time differences between the treatment (user fee abolished rural) regions and control regions. Additional controls in the model are described below.

The DID effect is estimated from the interaction term. It is a valid estimate of the user fee effect, if there is ‘baseline uniformity across time for the same region’ [33]. In other words, other time-varying processes are required to have similar impacts on both the treatment and comparison regions. If, for instance, the abolition of user fees was implemented in areas where facility-based deliveries were most responsive, validity would be threatened. Therefore, we check whether differences in characteristics in the treatment and control regions are systematically related to underlying changes in deliveries.

To investigate the assumption of a common time trend between the treatment and comparison groups in the absence of the reform, we perform a placebo test, where we pretend that the abolition of user fees took place in the pre-reform period, and examine control variables before and after the reform. Significant effects in the placebo tests would suggest that the estimated policy impacts reflect differential time trends, rather than true policy effects. In examining whether there are differences in the control variables, simple means tests were considered. Although there are signs of stability for some of the population characteristics, there are notable differences that emerge. To control for these observed differences between the comparison and treatment groups, we include interaction terms between some characteristics of the women and the policy reform variable. We also take steps to make sure that selective migration of women into the treatment and comparison areas is not driving the results by including, as already noted, a measure of residence duration.

## Results

### Descriptive statistics

Table 2 presents means and mean differences before and after the user fee abolition announcement, along with a test of significant differences within each group. Even before undertaking the analysis, the mean differences suggest limited effects. Across both groups, delivery at home is more likely after the announcement than before, while public facility delivery is less likely, and the estimated mean differences are quite similar across the groups. However, there are other significant differences across treatment and control groups, potentially violating the DID validity assumptions. Although there is some evidence of an increase in antenatal visits between the treatment and the control group, this difference is not significant. Possibly, due to the complementarity between the types of pregnancy-related care and giving birth in a health care facility, the increase in antenatal visits could be related to the removal of user fees for childbirth. However, antenatal care in Zambia has been provided for free for more than a decade and for the sub-sample used in this study, only 2 percent of women did not have any antenatal visits. Moreover, there are similar trends in antenatal visits within the control and the treatment groups, with both groups experiencing a significant decline in antenatal visits during the pre-reform and post reform period. In the subsequent sections, we consider additional sensitivity analyses.

**Table 2:** Comparison and treatment groups before and after the removal of user fees

Variable	Comparison group (N = 2118)			Treatment group (N = 1500)		
	Pre-reform	Post-reform	Diff (post–pre)	Pre-reform	Post-reform	Diff (post–pre)
Dependent variables						
Delivery at home	0.312	0.354	0.042***	0.566	0.607	0.041**
Delivery at a public health facility	0.646	0.612	−0.034*	0.373	0.340	−0.033*
Delivery at a private health facility	0.042	0.034	−0.0081	0.061	0.053	−0.0078
Independent variables						
*Individual level variables*						
Parity						
1	0.242	0.266	0.024	0.173	0.162	−0.011
2–4	0.486	0.454	−0.033	0.461	0.488	0.027
5+	0.271	0.280	0.009	0.367	0.350	−0.016
ANC utilisation						
Four or more visits	0.669	0.524	−0.144***	0.674	0.564	−0.110***
Religion						
Catholic	0.202	0.204	0.002	0.162	0.167	0.005
Protestant	0.786	0.787	0.000	0.822	0.815	−0.007
Other	0.011	0.009	−0.002	0.016	0.018	0.002
Household childcare burden						
Number of children under five in HH	1.467	1.877	0.410***	1.580	2.045	0.466***
Woman’s employment status						
Employed	0.521	0.438	−0.084***	0.554	0.467	−0.087***
Woman’s education						
No education	0.075	0.080	0.005	0.143	0.162	0.018
Primary	0.502	0.564	0.062**	0.649	0.632	−0.017
Secondary and above	0.423	0.356	−0.067***	0.207	0.206	−0.001
Partner’s education						
No education	0.164	0.146	−0.018	0.160	0.183	0.023*
Primary	0.318	0.383	0.066***	0.482	0.491	0.009
Secondary and above	0.518	0.470	−0.048**	0.357	0.326	−0.031*
Index for actual quality of ANC received	1.568	0.884	−0.683***	−0.591	−1.014	−0.423**
Household wealth						
Poorest	0.195	0.226	0.031*	0.518	0.561	0.043**
Middle	0.150	0.179	0.029*	0.272	0.274	0.002
Rich	0.655	0.595	−0.060***	0.210	0.165	−0.045***
Duration of residence						
0–4 (previous residence, rural)	0.167	0.204	0.037**	0.224	0.268	0.044***
0–4 (previous residence, urban)	0.291	0.269	−0.021	0.090	0.083	−0.007
5+	0.542	0.526	−0.016	0.686	0.649	−0.038**
*Community level variables*						
Area type						
Urban	0.655	0.599	−0.056**	0.178	0.151	−0.027*
Proportion of drugs available	0.726	0.739	0.013**	0.696	0.696	0.000
Material deprivation index	−1.633	−1.770	−0.137	0.512	0.545	0.033
Distance to nearest facility	3.775	4.374	0.600***	8.876	9.165	0.289
Density of health facilities	0.117	0.118	0.001	0.150	0.147	−0.004*
Prenatal care uptake	0.904	0.902	−0.002	0.988	0.988	0.001
TBA per 1000 of pop	0.345	0.350	0.005	0.524	0.526	0.002

Analysis variable means by treatment/control before and after the announcement of the abolition of delivery user fees in rural districts in Zambia, along with results from mean difference tests

* Significant difference at 0.05

** Significant difference at 0.01

*** Significant difference at 0.001

### User fee policy effect

As noted above, there are two levels in the multilevel framework applied here. Level 1 contains individual-level variables, while level 2 includes community-level factors. In our structure, individual and household characteristics are individual-level variables. The primary coefficient estimates are presented in Table 3. In keeping with the level description, Model 1 includes individual characteristics, only, while Model 2 includes both individual-level and community-level variables. The null model does not include either individual-level or community-level variables. The results are only available for the three variables essential to the DID specification: *D*_2006_ (the time dummy), *D*_*region*_ (region dummy) and *D*_2006_ × *D*_*region*_ (their interaction).

**Table 3:** Estimates of differences-in-differences user fee effects on location of birth. Estimates from three multilevel models reporting DID-specific coefficients only. Statistical results from ‘xtlogit’ estimated using Stata 12 (StataCorp 2011). Separate regressions undertaken for each birthing location. Standard error is robust to cluster-level random effects. Multinomial response models, available from the authors upon request, yield the same qualitative results

Variable	Home		Private		Public	
	Coefficient	Std. error	Coefficient	Std. error	Coefficient	Std. error
Null model: without controls						
Delivery after abolition of fees	0.114	0.138	−0.461	0.357	−0.072	0.131
Lives in one of the 54 districts with policy reform	1.485***	0.196	0.756*	0.458	−1.356***	0.183
DID estimator	−0.055	0.171	0.486	0.442	0.011	0.164
Model 1: with individual level controls						
Delivery after abolition of fees	−0.091	0.222	−0.537	0.540	0.216	0.206
Lives in one of the 54 districts with policy reform	−0.053	0.198	0.872	0.701	0.000	0.204
DID estimator	0.117	0.261	1.534**	0.660	−0.321	0.245
Model 2: with individual and community level controls						
Delivery after abolition of fees	−0.090	0.224	−0.681	0.556	0.226	0.208
Lives in one of the 54 districts with policy reform	−0.131	0.214	0.982	0.808	0.203	0.220
DID estimator	0.125	0.263	1.575**	0.671	−0.337	0.247

* Significant difference at 0.05

** Significant difference at 0.01

*** Significant difference at 0.001

The coefficients for the remaining variables are presented in Table 5, along with model performance information. Preferred models are those with lower absolute log-likelihood, Akaike Information Criterion (AIC) and Bayesian Information Criterion (BIC) values. Model 2, therefore, was preferred for explaining deliveries at home and deliveries in a public health facility. For private health facilities, on the other hand, Model 1 was preferred. Despite model preference, the qualitative individual-level effects reported in Table 5 do not differ across Model 1 and 2. The only difference lies in the community effects.

According to the DID estimates, user fee abolition did not have a statistically significant effect on delivery location choices for home and public deliveries, although a significant positive effect on deliveries in private or faith-based health facilities for rural Zambian women was uncovered. However, this last result needs to be interpreted with caution, as only about five percent of the sample had deliveries in private or faith-based health facilities. To provide additional context to this finding, 81 percent of health facilities in Zambia are government owned, 13 percent are private health facilities (the majority are in the urban areas) and six percent are owned by faith-based organisations. As indicated earlier, we combined the categories for faith-based and private deliveries, of which more than 95 percent are faith-based health facilities. In other words, the increase is driven by deliveries at faith-based facilities in rural areas. Furthermore, the policy did not have any significant effect on home deliveries or deliveries in public health facilities.

In an effort to be sure our results are not driven by model choice or the underlying differences reported in Table 2, further tests were undertaken. The DID approach requires common time trends across groups. Therefore, one concern is that these results reflect differential time trends. Specifically, the differential increase in fertility rates in rural areas over the five years prior to the survey, could possibly lead to an increase in births at the faith-based facilities in rural areas. To investigate, we estimate a placebo reform, Table 4, pretending that the abolition of user fees took place earlier than it did. The pre-reform sample is arbitrarily separated into two equal groups, and the intervention (timing) dummy is redefined to match the placebo. If there were differential time trends, the placebo reform estimate should be significantly different from zero; it is not, regardless of delivery location choice. Restricting the sample to include women who gave birth 2 years prior to the policy change (January 2004 to March 2006), on the basis that they may be less different than those who had given birth further in the past, also yields insignificant effects for the choice of location of delivery, except for deliveries in the faith-based facilities.

**Table 4:** Difference-in-difference sensitivity analysis. Estimates from multilevel models reporting DID coefficients only. Separate regressions undertaken for each birthing location and include different sets of controls and different policy announcement dates. The reported standard error is robust to cluster-level random effects

	Home		Private		Public	
	Estimate	S.E	Estimate	S.E	Estimate	S.E
Controls + selective migration						
DID estimator	0.125	0.263	1.575**	0.671	−0.337	0.247
Controls + facility density						
DID estimator	0.120	0.263	1.570**	0.672	−0.333	0.247
Controls + facility density interaction						
DID estimator	0.152	0.312	1.295	0.814	−0.365	0.300
DID estimator × facility density	−0.001	0.004	0.007	0.012	0.001	0.004
Controls + average antenatal interaction						
DID estimator	3.168**	1.303	0.294	3.619	−2.726**	1.224
DID estimator × average antenatal	−1.104**	0.462	0.443	1.239	0.864**	0.433
Controls + ancindex interaction						
DID estimator	0.150	0.267	1.521**	0.679	−0.357	0.251
DID estimator × ancindex	−0.067	0.117	−0.594	0.399	0.055	0.113
Controls + wealth interactions						
DID estimator	0.186	0.297	1.303*	0.790	−0.403	0.283
DID estimator × middle	−0.136	0.267	0.518	0.817	0.145	0.267
DID estimator × rich	0.273	0.748	0.355	1.122	−0.134	0.576
Controls + first birth						
DID estimator	0.218	0.265	1.652 **	0.673	−0.436*	0.249
DID estimator × first birth	−0.601*	0.358	−0.951	1.022	0.669*	0.352
Controls (less than secondary education + stable residence)						
DID estimator	0.063	0.258	1.324**	0.621	−0.587	0.359
Placebo reform (with controls)						
DID estimator	−0.105	0.336	1.330	0.809	−0.280	0.317
Controls +2 years pre-reform						
DID estimator	0.035	0.257	1.205**	0.613	−0.310	0.257

Another potential violation of the DID identification strategy is selective migration, i.e., expectant mothers relocate to take advantage of the reform. Thus, variables capturing previous residence are included in the regression models. However, it is also possible for a mother to continue to reside in an urban district, but give birth in a neighboring district, where fees may be lower, but not necessarily change residence. Although it is not possible to directly examine this possibility, we do so by interacting the DID effect with the first birth. In the Zambian set-up, women with first births may choose to deliver in an area where the mother resides, which may be in a rural area, and return to the matrimonial home after delivery. This analysis also produces insignificant effects for the choice of location of delivery, except for deliveries in the faith-based facilities.

A further concern is that the composition of the treatment and control groups may be changing pre and post-policy. Thus, it is possible that the DID effect may be reflecting changing composition rather than the policy effect. Most notably, from Table 2, it appears that the sample giving birth in the comparison group (urban districts) after the policy change is less educated, while higher levels of maternal education are associated with a higher probability of giving birth in a public health facility relative to home birth. Thus, the observed increase in home births among the comparison group may be reflecting such shifts. Although we control for education in the models, this may not be enough, if there is a differential education shift. Similarly, there is some evidence from Table 2 that migration patterns may also be different over this period. To address these concerns, we create a relatively homogeneous sample along the preceding dimensions, and conduct the analysis again. Specifically, we limit the sample to mothers with a primary education or below, whose partners also have only primary education or less and whose residence has been stable over the past five years. The DID effects for this sub-sample are as reported previously: no effect on home and public deliveries, but a positive effect for deliveries in faith-based facilities (see Table 4).

Even though we find no effect for home and public deliveries, that result may mask heterogeneity across women, who may respond differentially, due to their different contexts. For instance, women who live closer to a health facility or in an area with a higher density of health facilities may respond positively to the policy change. Also, mothers who had prior contact with the health staff at the facility are more likely to deliver at the health facility. Thus, we interact the DID dummy with variables, such as the distance to health facility, wealth, women whose delivery is for their first birth, the quality of antenatal care and average antenatal care uptake in the community. The interactions do uncover heterogeneities in the effect of policy change, which are driven by relative experience with the public health sector, that are averaged-out of the main analysis (see Table 4). In particular, communities with greater average antenatal care uptake experienced an increase in deliveries in public health facilities and a reduction in home deliveries. In addition, women with a first birth are significantly less likely to deliver from home but are more likely to deliver from a public health facility (see Table 4). Although it is expected that women with prior experiences may be more likely to deliver from a health facility, based on the quality of care provided, we find no significant effect after interacting the DID estimator with the measure of quality of delivery services. With respect to the remaining potential heterogeneous effects, none were uncovered for wealth, selective migration, or even facility density. Furthermore, the positive effect of the removal of user fees on deliveries at faith-based health facilities persists.

To further control for time-invariant characteristics at the area level, we also run regressions incorporating district or cluster fixed effects. The results obtained are similar to the main regression results.

## Discussion

### Main effects

We find no evidence that user fee abolition in the 54 rural Zambia districts affected, on average, home or public facility deliveries, although deliveries in faith-based facilities increased. However, we find heterogeneous impacts of the policy reform on home deliveries in communities with low antenatal care uptake and on public deliveries in communities that have higher uptake of antenatal care services. The main results are robust to a series of specification checks, but limitations remain. While the 2005 ZHFC contains information on health facilities collected at one point in time, the health facility information could have changed, affecting mother’s choices. However, health facilities data enabled us to include measures not captured in the household survey, such as distance and drug availability. Unfortunately, it is only possible to measure distance in a straight line to the nearest facility, and not necessarily to facilities providing delivery services. While 93% of the facilities covered in the ZHFC provide ANC services [27], not all of them provide delivery services; bias could, therefore, be generated by including all the facilities. As is true with all DHS data analysis, the sample does not include women who died, due to child birth-related complications. Also, data from routine health information systems can be problematic. However, few outliers were detected in our analysis of the HMIS data. Moreover, at the national level, about 92% of HMIS reports from health facilities were complete in 2007 indicating that the data was of good quality. Another potential limitation is that data from private health facilities is not generally incorporated into the HMIS, and therefore, the data might not reflect all health facilities in the country.

It is also important to highlight two other caveats. The first is that the data was collected retrospectively, and, therefore, might not entirely reflect the circumstances the women were under at the time of birth. Although it is not possible to correct this with the data that is available, the specification tests reported in Table 4 suggest that our results are not driven by that limitation. Neither altering, via a placebo experiment, nor including residential duration are found to alter our conclusions. The second is that, although the treatment and control group sample sizes are both fairly large, the treatment group only represents births recorded in the year immediately following the policy announcement. It is possible that the policy, despite being announced, was not completely internalized by either the health districts or expectant mothers. In other words, the data that is available could be too early to detect effects on deliveries in public facilities, and, therefore, it is recommended that this research be extended to include future ZDHS waves [13].

### Policy interpretation

Despite the caveats and lack of user fee abolition effects on deliveries in public facilities and on home deliveries, the findings matter for policy. Abolishing user fees was expected to increase maternal health service utilisation in public facilities, as it has increased the utilisation of other health services in Zambia and other developing countries [8, 9, 15]. While user fees determine the direct cost of delivery services, there are also indirect costs. Our other estimates in Table 5 suggest that indirect costs could be important [34, 35].

**Table 5:** Complete multilevel estimates of the effect of user fee abolition on location of childbirth. Estimates from three multilevel models reporting DID-specific coefficients only. Statistical results from ‘xtlogit’ estimated using Stata 12. Separate regressions undertaken for each birthing location. Standard error is robust to cluster-level random effects. Multinomial response models, available from the authors upon request, yield the same qualitative results

	Model 1						Model 2					
	Home		Private		Public		Home		Private		Public	
	Estimate	S.E	Estimate	S.E	Estimate	S.E	Estimate	S.E	Estimate	S.E	Estimate	S.E
*Individual level variables*												
Delivery post abolition of user fees	−0.091	0.222	−0.537	0.540	0.216	0.206	−0.090	0.224	−0.674	0.556	0.226	0.208
Lives in one of the district with policy change	−0.053	0.198	0.872	0.701	0.000	0.204	−0.131	0.214	0.830	0.821	0.203	0.220
DID estimator	0.117	0.261	1.534**	0.660	−0.321	0.245	0.125	0.263	1.568**	0.671	−0.337	0.247
Parity (ref = 1)												
2–4	0.701***	0.202	−0.111	0.473	−0.552***	0.190	0.744***	0.202	−0.159	0.470	−0.581***	0.190
5+	0.860***	0.212	0.438	0.519	−0.783***	0.202	0.915***	0.213	0.395	0.516	−0.811***	0.202
ANC utilisation												
Four or more visits	−0.313**	0.123	0.221	0.338	0.243**	0.120	−0.329***	0.124	0.194	0.338	0.262**	0.120
Religion (ref = Catholic)												
Protestant	0.020	0.162	0.000	0.441	−0.071	0.158	0.006	0.161	−0.077	0.438	−0.056	0.157
Other	0.708	0.794	2.961*	1.554	−0.770	0.729	0.675	0.787	3.098**	1.534	−0.831	0.726
Household childcare burden												
Number of children under five in HH	0.067	0.070	0.094	0.195	−0.069	0.068	0.059	0.071	0.091	0.195	−0.063	0.069
Woman’s employment status (ref = unemployed)												
Employed	−0.327***	0.126	0.782**	0.344	0.179	0.121	−0.328***	0.126	0.710**	0.343	0.190	0.121
Woman’s education (ref = no education)												
Primary	−0.494***	0.177	−0.038	0.604	0.522***	0.178	−0.492***	0.177	0.039	0.601	0.533***	0.178
Secondary and above	−0.744***	0.234	0.308	0.719	0.594***	0.229	−0.734***	0.234	0.348	0.718	0.601***	0.229
Partner’s education (ref = no education)												
Primary	0.103	0.219	−0.867	0.646	−0.010	0.218	0.142	0.218	−0.926	0.635	−0.041	0.217
Secondary and above	0.021	0.240	−1.241*	0.689	0.060	0.238	0.087	0.240	−1.229*	0.677	0.016	0.238
Index for actual quality of ANC received	0.397***	0.060	−0.340*	0.205	−0.325***	0.059	0.367***	0.060	−0.375*	0.204	−0.299***	0.058
Household wealth (ref = poorest)												
Middle	−0.325**	0.137	−0.268	0.462	0.347**	0.140	−0.206	0.143	−0.239	0.474	0.260*	0.144
Rich	−2.615***	0.300	0.970	0.798	1.917***	0.270	−1.887***	0.377	1.399	0.996	1.146***	0.336
Duration of residence (ref = previous res, rural)												
0–4 (previous residence, urban)	−0.207	0.246	0.566	0.660	−0.026	0.225	−0.210	0.248	0.620	0.658	−0.059	0.225
5+	0.413***	0.144	1.087**	0.552	−0.501***	0.143	0.403***	0.144	1.132**	0.553	−0.505***	0.143
*Community level variables*												
Area type (ref = rural)												
Urban							−1.004***	0.242	−0.660	0.852	1.032***	0.247
Material deprivation index							−0.034	0.652	3.382	2.464	−0.530	0.687
Distance to nearest facility							0.208**	0.095	0.040	0.297	−0.175*	0.096
Density of health facilities							−0.024*	0.013	0.035	0.050	0.023	0.014
Prenatal care uptake							−0.189	0.266	2.531***	0.884	−0.197	0.270
TBA per 1000 of pop.							0.000	0.001	−0.001	0.002	0.001	0.001
Proportion of drugs available							0.992***	0.311	0.431	1.117	−0.914***	0.327
Constant	0.165	0.393	−6.224***	1.399	−0.180	0.393	0.139	1.004	−15.704***	3.764	1.054	1.033
Rho	0.122		0.674		0.18		0.097		0.625		0.146	
LR test (Chi2 value)	25.28***		94.1***		54.3***		18.01***		64.3		38.5***	
Log likelihood	−1010.1		−261.5		−1126.9		−985.67		−249.21		−1099.34	
AIC	2062.2		564.9		2295.8		2027.33		554.41		2254.68	
BIC	2178.9		669.7		2413.5		2182.81		694.04		2411.59	
Observations	1912		1086		2013		1906		1082		2006	

* Significant difference at 0.05

** Significant difference at 0.01

*** Significant difference at 0.001

Increased distance to the health facility increases home delivery and decreases facility delivery [36]. Other indirect costs include food and lodging for the expectant mother and any accompanying relatives, especially if the woman is admitted for in-patient care [18]. Since women in rural areas have to cover longer distances to get to the facility, compared to their urban counterparts, the construction of waiting shelters should be encouraged, in order to accommodate birthing women, and, possibly, their accompanying relatives.

Even though user fees did not provide a significant source of income for health facilities, they were a flexible form of income and were used by health facilities in rural areas to support the functions of TBAs, mostly in the form of tokens of appreciation for encouraging women to deliver at health facilities [29]. Other uses included the buying of cleaning agents (bleach) and food for inpatients. However, with the abolition of user fees, this support to TBAs has significantly reduced, as has the incentive for them to encourage woman to deliver at health facilities. Qualitative studies from the Eastern province in Zambia show that, in some cases, women were required to bring bleach, soap, nappies, a baby blanket, Vaseline, a baby suit and *chitenge*—a wrapper used by women [37]. This shifting of costs erects a new barrier to the utilisation of delivery services in place of the user fee barrier. Women who struggle to provide these items may decide, instead, to deliver at home. Providing expectant mothers with baby layettes—a package containing basic requirements for a new born baby—as is done in some health facilities in Zambia, could encourage the utilisation of delivery services. Women without them may fear they will be ostracised.

Given the aforementioned indirect costs, rural women may seek care from traditional birth attendants (TBAs), who are often located within the communities and have negotiable payment terms that are rather low [16]. In rural areas, TBAs are trained to carry out deliveries and are advised to refer more complicated cases to higher levels of care. A larger share of women were assisted by TBAs in 2007 (23.5%) compared to 2001/2 (11.7%) [1]. Thus, the involvement of trained TBAs during delivery remains an important component of delivery in Zambia, particularly in rural areas with few health workers [28]. However, we do not find that the presence of TBAs in the community affects expectant mother decisions.

The association between ANC take-up and delivery decisions calls for the continued promotion of ANC attendance. Although it is evident that, ultimately, a reduction in maternal deaths may be brought about through adequate delivery care, often available only at health facilities, our results further support existing evidence that ANC is key in encouraging facility-based deliveries [7]. Similarly, the mother’s education is found to be important.

The most surprising finding concerns the role of our quality of care measures in shaping the utilisation of facility deliveries. Marking a clear departure from previous qualitative literature [16], the index of care quality, the density of health facilities and drug availability are all found to be negatively associated with delivery in public health facilities, which is offset by increases in home deliveries. However, with respect to the quality index, there is some evidence that ANC quality raises the relative likelihood of public facility usage, relative to private facility deliveries. Possibly, women report better quality of care in private health facilities, even though the cost deters them from using the service [38]. One explanation for our surprising result is that, although overlap is expected, it is possible that there are differences between unobserved perceived quality and our measure, which may only be weakly linked to better quality [39]. The perception of quality largely depends on individuals’ experiences with the health system or that of users known to them [40], and may not relate to whether or not the proper procedures were undertaken during ANC visits.

We find that women in urban areas and less deprived areas are more likely to deliver in health facilities, which is consistent with previous literature [19]. Even though, the link between wealth and facility-based delivery might be confounded by the correlation between area of residence and financial resources, the result is as expected: wealthier women are more likely to use (public) health facilities, and that may relate to the ability to reach good quality health facilities [41]. In other words, transport costs and opportunity costs are lower for those who live closer to facilities, and they are also likely to be wealthier. Possibly, mitigating the link between socioeconomic status and the use of delivery services could be brought about by empowering communities, and putting them at the centre of their own development, which they might use to develop community-led emergency transport systems [42].

## Conclusion

This study investigates the effect of the abolition of user fees on the place of delivery using a multilevel DID framework. By analysing both individual- and community-level effects, this analysis goes beyond the evaluation of the policy change. Although understanding the effect of policy is important, understanding the main determinants of the place of delivery improves our understanding of the link between health care policies and birthing womens’ demands for delivery services.

The findings indicate that user fee abolition was not sufficient to increase facility-based deliveries at public health facilities, on average, although we find evidence of an increase in utilization in areas where ANC uptake was higher than average. Although simple economic reasoning suggests that the removal of a price barrier should lead to an increase in utilisation of health services, economic behaviour can be more complicated. The elimination of a price barrier (on demand), which is also a revenue (for suppliers), may lead to cost-shifting, resulting in an indirect non-price barrier. Furthermore, price effects are also likely to relate to social norms, such that higher acceptance of the public health sector, as evidenced by higher average ANC uptake, shows a greater willingness to take advantage of the sector, when its direct costs fall. Overall, the findings underline the importance of indirect costs in deterring birthing women from seeking delivery services at a health facility. To ensure that women, currently not seeking delivery services at a health facility, gain access, policies to address non-financial barriers are needed, as are policies to further improve the acceptance of ANC. A detailed analysis of the indirect costs incurred by the households during delivery could not be studied at this stage, due to data inadequacies, but should be considered for future research.

## Abbreviations

ANC: Ante Natal
DID: difference-in-differences
HMIS: Health Management and Information Health System
MDI: material deprivation index
MoH: Ministry of Health
MCA: multiple correspondence analysis
PCA: principal component analysis
TBA: traditional birth attendants
ZDHS: Zambia Demographic and Health Survey
ZHFC: Zambia Health Facility Census
